# Wireless Body Area Network for Preventing Self-Inoculation Transmission of Respiratory Viral Diseases

**DOI:** 10.3390/s23042042

**Published:** 2023-02-11

**Authors:** Łukasz Pawlicki, Alicja Fotyga, Jakub Rewieński, Mateusz Groth, Łukasz Kulas, Grzegorz Fotyga

**Affiliations:** Faculty of Electronics, Telecommunications, and Informatics, Gdansk University of Technology, 80-233 Gdansk, Poland

**Keywords:** wireless body area network, wearable medical sensors, machine learning applications, epidemic, health

## Abstract

This paper proposes an idea of Wireless Body Area Networks (WBANs) based on Bluetooth Low-Energy (BLE) standards to recognize and alarm a gesture of touching the face, and in effect, to prevent self-inoculation of respiratory viral diseases, such as COVID-19 or influenza A, B, or C. The proposed network comprises wireless modules placed in bracelets and a necklace. It relies on the received signal strength indicator (RSSI) measurements between the bracelet and necklace modules. The measured signal is cleared of noise using the exponential moving average (EMA). Next, we use a classification algorithm based on a Least-Squares Support Vector Machine (LSSVM) in order to detect facial touches. When the results of the classification indicate that the hand is moving toward the face, an alarm is sent through the neck module and the vibrator embedded in the wrist module is switched on. Based on the performed tests, it can be concluded that the proposed solution is characterized by high accuracy and reliability. It should be useful, especially for individuals who are regularly exposed to the risk of respiratory infections.

## 1. Introduction

The severe acute respiratory syndrome coronavirus 2 (SARS-CoV-2) outbreak started in Wuhan, Hubei province, China, in November 2019. Since this disease is highly infectious, it spread rapidly all over the world, eventually reaching the necessary epidemiological criteria for being recognized as a pandemic on 11 March 2020 [1]. It has affected many areas of the economy as well as everyday life, leading to severe global disruption.

Similarly to other respiratory viral diseases, the virus can be transmitted during close and unprotected contact between an infector and infectee, as reported in [2]. People can also become infected by touching surfaces in public spaces (such as handles, payment terminals, and taps) contaminated with pathogens. This issue is especially significant since it has been reported recently that SARS-CoV-2 can survive on plastic and stainless steel for up to 72 h after application to these surfaces [3]. Next, the infection can be caused by the self-inoculation process [4,5,6,7,8], in which contaminated hands can transmit respiratory infections by touching a face, especially nose, mouth, or eyes. Taking into account that, according to [4], people touch their face approximately 24 times per hour. This habit can significantly contribute to the spread of the pandemic process, even if it can be limited, to some extent, by wearing masks, according to the results published in [8,9,10,11].

An obvious recommendation provided by the World Health Organization (WHO) to prevent self-inoculation of respiratory viral diseases it to wash hands frequently [12]. An interesting method for monitoring hand hygiene activity among healthcare workers, using head-mounted cameras, has been proposed in [13]. The review of the hand hygiene compliance monitoring methods is published in [14], whereas in [15], the authors propose a smart home system based on radars, which is able to detect such activities as coughing, sneezing, and face touching in order to prevent the spreading of disease.

However, in this paper, we focus on solutions related to wireless body area networks (WBAN). Recently, a few wearable systems to track and detect facial touch gestures have been proposed in the literature. The system described in [16] utilizes an inertial measurement unit (IMU) equipped with an accelerometer and gyroscope to obtain features that characterize hand movement, such as face touching. Time-series data obtained from IMU is classified using a 1D-Convolutional Neural Network (CNN), Recurrent Neural Network (RNN) [17], or decision tree, k-nearest neighbor, and support vector machine [18]. Similarly, in [19,20,21], accelerometer data collected from the wrist position (a smartwatch application) has been used to generate machine-learning models to recognize facial touches. Papers [22,23] propose a system called *No Face-Touch*, which is able to estimate hand proximity to face and notify the user whenever a face-touch movement is detected. The system consists of a smartwatch with inertial and magnetic sensors and a necklace with five magnets. Finally, the approach proposed in [24] is based on the ear-worn system capable of identifying actual touches of the face, using low-resolution thermal images and physiological signals (impedance changes caused by skin deformation during a touch). Next, these signals are fed into a deep-learning model.

In this paper, an alternative method of the hand-gestures classification is proposed to detect facial touch gestures and, in consequence, to prevent self-inoculation of diseases. To the best of the authors’ knowledge, similar solutions have not yet been published in the literature. It relies on the WBAN, which comprises the three modules (nRF52840 [25]) placed on the wrists (in the form of bracelets) and one placed on the neck (as a necklace). Contrarily to the systems proposed in [16,17,18,19,20,21,22,23,24] m,face-touching gestures are recognized by analyzing the received signal strength indicator (RSSI) between the neck and wrist modules, which are connected using the Bluetooth Low-Energy (BLE) network [26]. The classification process based on RSSI data is performed using the Least Square Support Vector Machine (LSSVM) algorithm [27]. Finally, tests of the proposed system show that the proposed method achieves a very high precision (at the 93% level). If the face-touching gesture is recognized, a vibrator and/or buzzer placed in the wrist module are switched on. It is expected that the proposed solution can be especially useful for communities that are regularly exposed to the risk of respiratory viral disease infections, more precisely, working in such areas as health care, public transport, or grocery trading.

## 2. Methods

In this section, we will describe the proposed gesture classification wireless system. However, before we get to the description of the system, we will look at the research devoted to the habit of touching the face with the hands. The analysis of this phenomenon is crucial to the design of a suitable WBAN architecture, together with proper signal analysis.

### 2.1. Face Touching

As mentioned in Section 1, face-touching is a potential vector for the self-inoculation of respiratory viral infections, such as COVID-19. In order to analyze face-touching habits, an interesting behavioral observation has been performed and reported in [4], in which the face-touching behavior of medical students was observed via videotape recording. The results of the observation are summarized in Table 1, where $n_{T}$ and $t_{D}$ denote the average number of face-touching per hour and average duration range, respectively. It can be seen that the average number of touches per hour is 24. The face parts are divided into mucosal areas (eyes, nose, and mouth, 44% of all touches) and non-mucosal areas (ears, cheeks, chin, forehead, and hair, 56% of all touches), where the former ones can potentially cause a self-inoculation.

Another interesting study related to face-touching frequency has been reported in [28]. It has been performed using video cameras mounted in the train. The average face-touching frequency was 17.8 times per hour, where the mucosal and non-mucosal contacts were at the levels of 42.2% and 57.8%, respectively. As can be seen, the results obtained in [28] are well correlated with [4].

The results summarized above show that the face-touching habit is a serious issue that can potentially cause self-inoculation of respiratory viral diseases, specifically when hands had had contact with surfaces contaminated with pathogens in public places, such as public transportation, hospitals, schools, etc.

### 2.2. Wireless Body Area Network for Preventing Face-Touching Habits

WBAN is represented by a set of sensors attached to the clothes or placed in or on the human body. It can be used in such applications as, for example, performance and wellness monitoring, sport, emergency, entertainment, and authentication [29]. Health-related WBAN networks are used to measure different medical parameters, such as body temperature, blood sugar level, heart/pulse rate, oxygen saturation, electrocardiogram (ECG), and Parkinson’s disease (an overview of WBAN’s medical files can be found in [30] and [29]). Sensors are connected using short-range wireless technologies, where the most popular communication standards are: Bluetooth, Bluetooth Low-Energy, ZigBee, and IEEE 802.15.6 [29]. Depending on the wireless technology used, the network may have a star, mesh, or tree topology.

In this article, we focus on a medical application that deals with the prevention of touching the face with the hands and, as a result, not contracting diseases. Taking into account the aspects described in the previous section, the hands that are touching the face are placed in a similar, upward position and the wrists are placed close to the neck. This observation is a good background for developing a WBAN, which would effectively recognize the face-touching movements.

**Figure 1:**
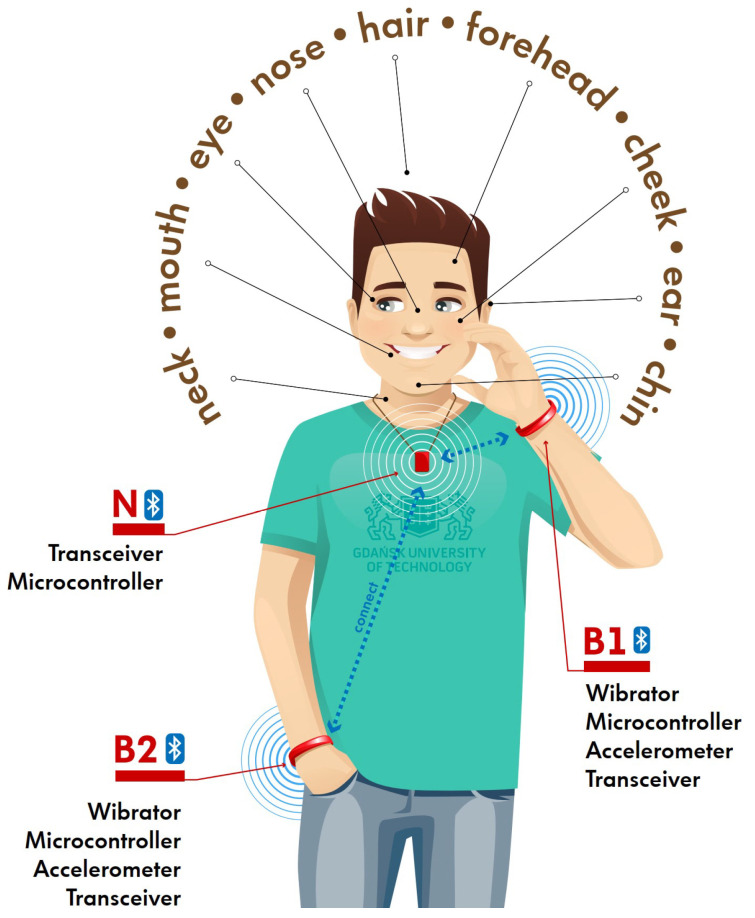
The scheme of the proposed WBAN system with a star topology. It consists of two BLE wireless modules placed on the wrists (B1 and B2) and one module placed on the neck (N).

The idea of a proposed WBAN system is shown in Figure 1. The network comprises:

- two bracelets (B1 and B2) containing the body area network modules equipped with a microcontroller, transceiver, and vibrator (buzzer). Alternatively, the system can comprise just one bracelet placed on the dominant wrist.
- necklace (N) with a BAN module equipped with a microcontroller and transceiver. It can be combined with other neck sensors, such as the ones described in [31,32,33,34].

The proposed system represents the star topology, where the B1 and B2 nodes are connected to a central coordinator (N). These modules can form a separate wireless system or be part of a larger general-purpose health-related system. The hand movements can be recognized using the accelerometers and/or gyroscopes placed in the B1 and B2 modules, similarly as in the solutions described in [20,21,35,36,37,38]. However, the accuracy of this approach is questionable. The signals collected by the accelerometers and gyroscopes strongly depend on the speed of hand movement, the position of a body, initial position of hands, movement time, etc.

Thus, in this work, we take into account an alternative approach, which is the received signal strength indicator (RSSI) measurements between the neck and the wrist modules (B1 and B2). These modules detect the movement of hands based on signals obtained from accelerometers. When movement is detected, a signal is transmitted from the modules on the wrists. This signal is received in module N (on the neck), and the RSSI of the signal is measured. The collected data are used next to classify gestures (in an external device, such as a mobile phone or in the N module) and, in effect, to detect face touching. Once the RSSI measurements indicate that the hand is moving toward the face (based on the classification process), an alarm is sent through the neck module, and the vibrator embedded in the wrist module is switched on.

In our tests, we used relatively cheap, small-size nRF52840 wireless modules [25], equipped with ARM Cortex-M4F processor, 1 MB/256 kB flash and RAM memory, respectively, which makes it suitable for quick computation involving floating point math. It allows for a high-speed data transfer (2 Mbps) and a low power mode current consumption.

An important advantage of the module is that it supports Bluetooth Low-Energy (BLE) communication technology. It means that it consumes approximately 90% less power compared to standard Bluetooth. This feature contributes to a significant increase in the life of the batteries powering the modules. Another advantage is that the synchronization between modules is much shorter than with Bluetooth, taking only a few milliseconds [30]. It should be noted that BLE is a standard system for such applications as the Internet of Things (IoT), advanced wearables, and interactive entertainment devices since it is characterized by considerably reduced power consumption without sacrificing a communication range. For this reason, BLE is a suitable standard for our application.

**Figure 2:**
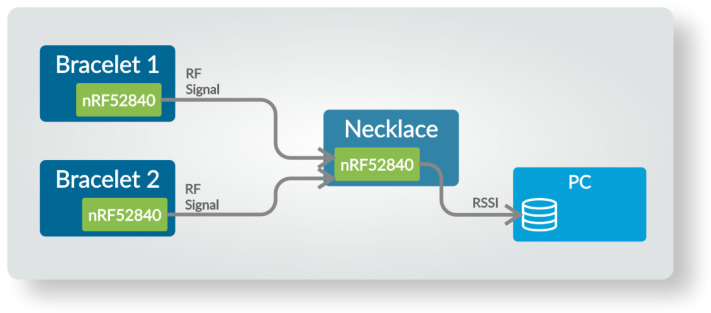
WBAN system architecture. It consists of two BLE wireless modules placed on the wrists (Bracelet 1 and Bracelet 2) and one coordinator module placed in a necklace. The measured RSSI signals can be classified externally (in a mobile phone or PC) by the coordinator.

The architecture of the system is presented in Figure 2. In the initialization phase of the system, the wireless modules (B1 and B2) connect to the coordinator module installed in the necklace (N), creating a star-topology WBAN system. Next, in the standard operation phase, module N is configured to measure the RSSI values of the packets received from modules B1 and B2. When movement of the B1 or B2 module is detected based on the signals obtained from accelerometers, a signal is transmitted from B1/B2 module to N. Then, the measured data are transferred to the PC and stored in the database for further signal analysis based on the classification algorithm. It should be noted that in the target WBAN system, it is possible to carry out signal classification in the central module (coordinator—N) or on the mobile phone to which the proposed system would be connected.

### 2.3. Analysis of Gesture-Related RSSI Measurements

Next, the obtained data were used to perform the analysis of the relation between gestures made by the user and the measured RSSI values. We assumed the signal power; 8 dBm, sampling rate/messaging rate; 100/3 Hz, each signal contained 100 samples. The gestures were divided into two groups, the face-touching ones (denoted by FT), namely, touching of the:

- nose
- eye
- ear
- lips
- hair

and other gestures (denoted by nFT):

- hand movement during standard walk
- opening the doors
- putting a book on the shelf
- shoulder scratching
- elbow scratching
- hand waving ’bye’.

Each of them was measured in different body positions and at different speeds, resulting in a set of 100 RSSI signals (50 FT and 50 nFT).

**Figure 3:**
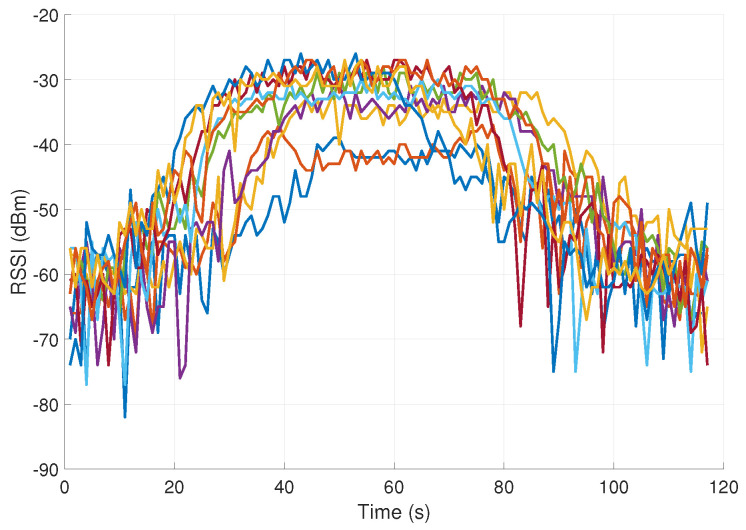
RSSI signals in dBm measurements associated with mouth touching (10 different signals representing the same hand movement kind).

**Figure 4:**
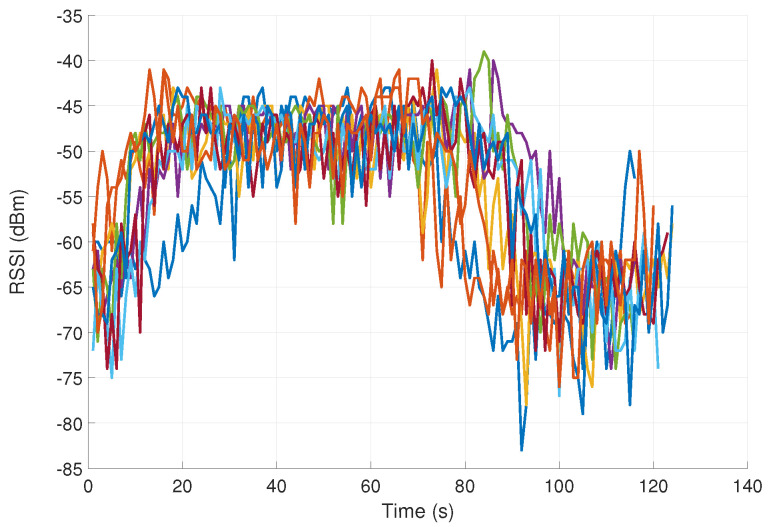
RSSI signals in dBm measurements associated with elbow scratching (10 different signals representing the same hand movement kind).

**Figure 5:**
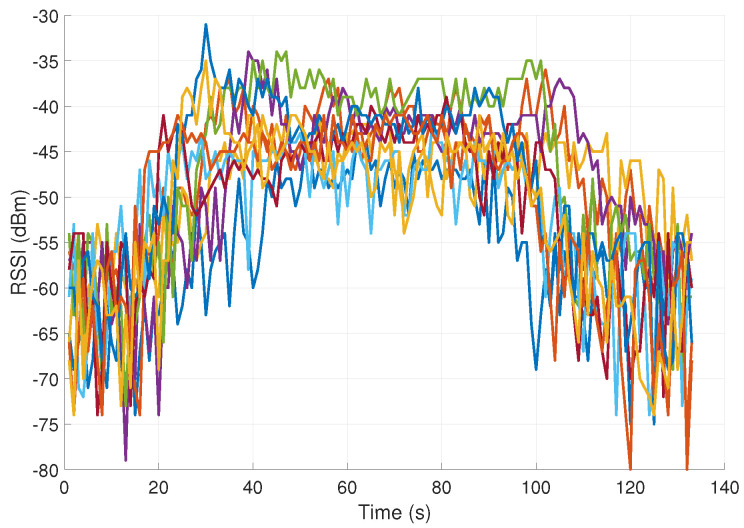
RSSI signals in dBm measurements associated with shoulder scratching (10 different signals representing the same hand movement kind).

Three examples of RSSI signal measurements (associated with mouth touching and elbow and shoulder scratching) are shown in Figure 3, Figure 4 and Figure 5. Each of the figures contains 10 different signals of the same kind (representing the same hand movement kind). As can be seen, the gestures vary in duration and dynamics. As expected, the FT gestures result in a considerably higher pick level of RSSI signal, as compared to nFT gestures.

It can be seen that the measured plots are distorted in the sense that two adjacent samples often have values that differ significantly from each other in magnitude. Such noise can significantly affect the classification process. In order to attenuate the high-frequency components in signals (remove high-frequency noise), we used the exponential moving average (EMA), described in [39]. It acts as a low-pass filter and is especially useful for analyzing rapidly changing signals.

**Figure 6:**
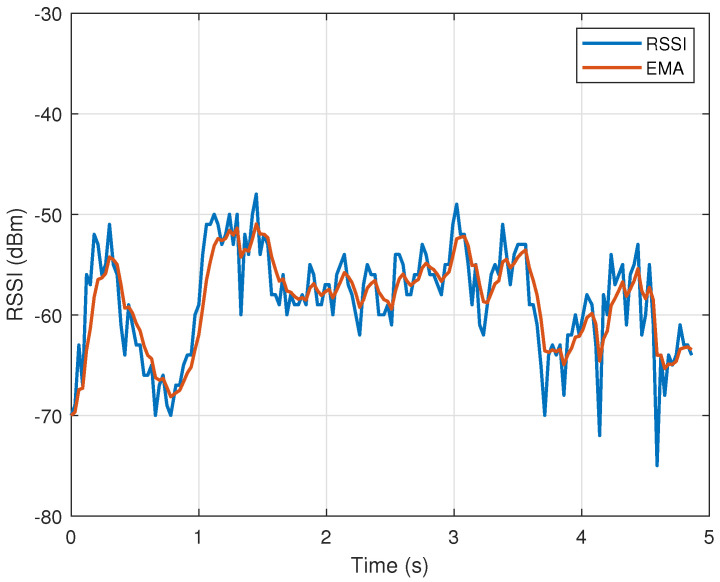
RSSI signal associated with elbow scratching. Blue line—RSSI measurements, red line—exponential moving average of measurements.

**Figure 7:**
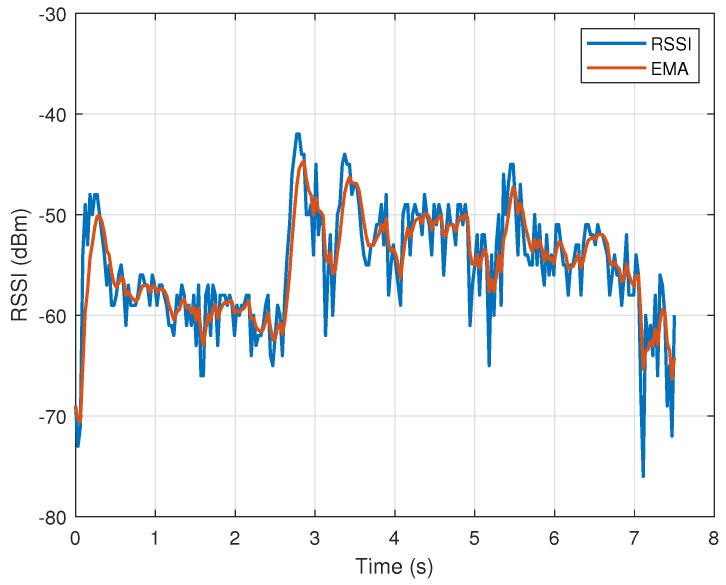
RSSI signal measurements associated with putting a book on a shelf. Blue line—RSSI measurements, red line—exponential moving average of measurements.

The recursive EMA formulation is defined as follows:

(1) $$s_{t}=\left\{\begin{array}{cc} y_{1}\;\;\;\;\; & t=1\\ \alpha y_{t}+\left(1-\alpha \right)s_{t-1}\;\;\;\; & t>1 \end{array}\right.$$

where *y* and *s* are the measured RSSI and EMA signals, respectively, and α=2/(n+1). EMA places a greater weight and significance on the most recent data points. The tests showed that the highest classification accuracy is obtained for *n* equal to 5. The results of the signal smoothing using EMA can be seen in Figure 6 and Figure 7. It can be seen that higher-frequency components are suppressed (EMA signals are much smoother compared to measurements).

### 2.4. Signal Classification

Next, the collected RSSI measurements were used to develop the machine learning model and to perform binary classification of the time-domain signals (to differentiate between FT/nFT gestures). Various algorithms can be used to classify time-series signals (K-Nearest Neighbors (KNN), Decision Trees, Random Forest, Naive Bayes, Neural Networks, and Support Vector Machine (SVM)). The best algorithm (in terms of speed and accuracy) for a particular signal classification task depends on the nature of the signals and the desired result. The choice of algorithm may also depend on factors such as the size and complexity of the dataset, available computing resources, and the desired classification accuracy and speed. However, the SVN algorithm has been used for this purpose since it allows for a high-speed and accurate classification of such time-domain signals, according to [40,41].

To this end, we used a Least Square Support Vector Machine (LSSVM) [27] library with radial basis function kernel, multidimensional unconstrained non-linear optimization (simplex) in the tuning stage, misclassification measure of residuals, and model performance estimation with 10-fold cross-validation. We obtained the following results:

(2) $$p=\frac{tp}{tp+fp}=0.93$$

(3) $$r=\frac{tp}{tp+fn}=0.94$$

(4) $$f1=\frac{2pr}{p+r}=0.94$$

where *p*, *r*, and f1 are precision, recall, and F-score performance metrics, respectively, and tp, fp, and fn are true-positive, false-positive, and false-negative results of binary classification, respectively. The obtained results show that the proposed BLE network allows for the efficient classification of FT and nFT gestures. It should be noted that p=0.93 accuracy is a comparable level to the algorithms discussed in [16,17,18,19]. This accuracy is quite sufficient for this type of application (taking into account the classification accuracies of 0.926, 0.90, and 0.97 in [18,22,24], respectively).

## 3. Conclusions

The purpose of this paper is to describe a new method for the prevention of self-inoculation of respiratory viral diseases, such as COVID-19 and influenza A, B, and C. For this purpose, a wireless body area network has been created, which consists of modules placed on the neck and wrist. The RSSI between the two modules is measured. The test showed that the RSSI classification using the LSSVM algorithm can effectively detect facial-touching gestures and thereby contribute to preventing the spread of respiratory viral diseases. However, it should be clearly stated that the presented system is at the proof of concept level. The performed experiments largely confirm its feasibility and reliability. However, further research needs to be performed to bring it to the level of a full commercial realization. In particular, the system should be optimized in terms of the costs of individual components, module sizes, power consumption, as well as user experience (UX) aspects. Further research should also be conducted to determine the most optimal (in terms of speed, memory usage, and accuracy) machine learning algorithm for gesture classification. 

## Figures and Tables

**Table 1:** The result of the face-touching habit observation reported in [4], where $n_{T}\left(s\right)$ and $t_{D}\left(s\right)$ denote the average number of face-touching per hour and average duration range in sec., respectively.

Area	$n_{T}\left(s\right)$	$t_{D}\left(s\right)$
mouth	4	3
eye	3	1
nose	3	1
hair	4	3
ear	1	3
cheeck	4	5
neck	1	5
chin	4	4
face	24	3.08

## Data Availability

Not applicable.
